# The fibroblast: An emerging key player in thymic T cell selection

**DOI:** 10.1111/imr.12985

**Published:** 2021-06-06

**Authors:** Takeshi Nitta, Ayami Ota, Takahiro Iguchi, Ryunosuke Muro, Hiroshi Takayanagi

**Affiliations:** ^1^ Department of Immunology Graduate School of Medicine and Faculty of Medicine The University of Tokyo Tokyo Japan

**Keywords:** Thymus, fibroblast, T cell, capsule, medulla

## Abstract

Fibroblasts have recently attracted attention as a key stromal component that controls the immune responses in lymphoid tissues. The thymus has a unique microenvironment comprised of a variety of stromal cells, including fibroblasts and thymic epithelial cells (TECs), the latter of which is known to be important for T cell development because of their ability to express self‐antigens. Thymic fibroblasts contribute to thymus organogenesis during embryogenesis and form the capsule and medullary reticular network in the adult thymus. However, the immunological significance of thymic fibroblasts has thus far only been poorly elucidated. In this review, we will summarize the current views on the development and functions of thymic fibroblasts as revealed by new technologies such as multicolor flow cytometry and single cell–based transcriptome profiling. Furthermore, the recently discovered role of medullary fibroblasts in the establishment of T cell tolerance by producing a unique set of self‐antigens will be highlighted.

## 1 INTRODUCTION

The thymus is an organ in which T cells develop and their antigen recognition repertoire is established.^1^ In the three‐dimensional microenvironment composed of thymic stromal cells, immature T cells (called thymocytes) undergo stepwise developmental processes, including differentiation, proliferation, and cell fate determination in order to give rise to mature T cells expressing a diverse T cell receptor (TCR) repertoire.^2^

**FIGURE 1:**
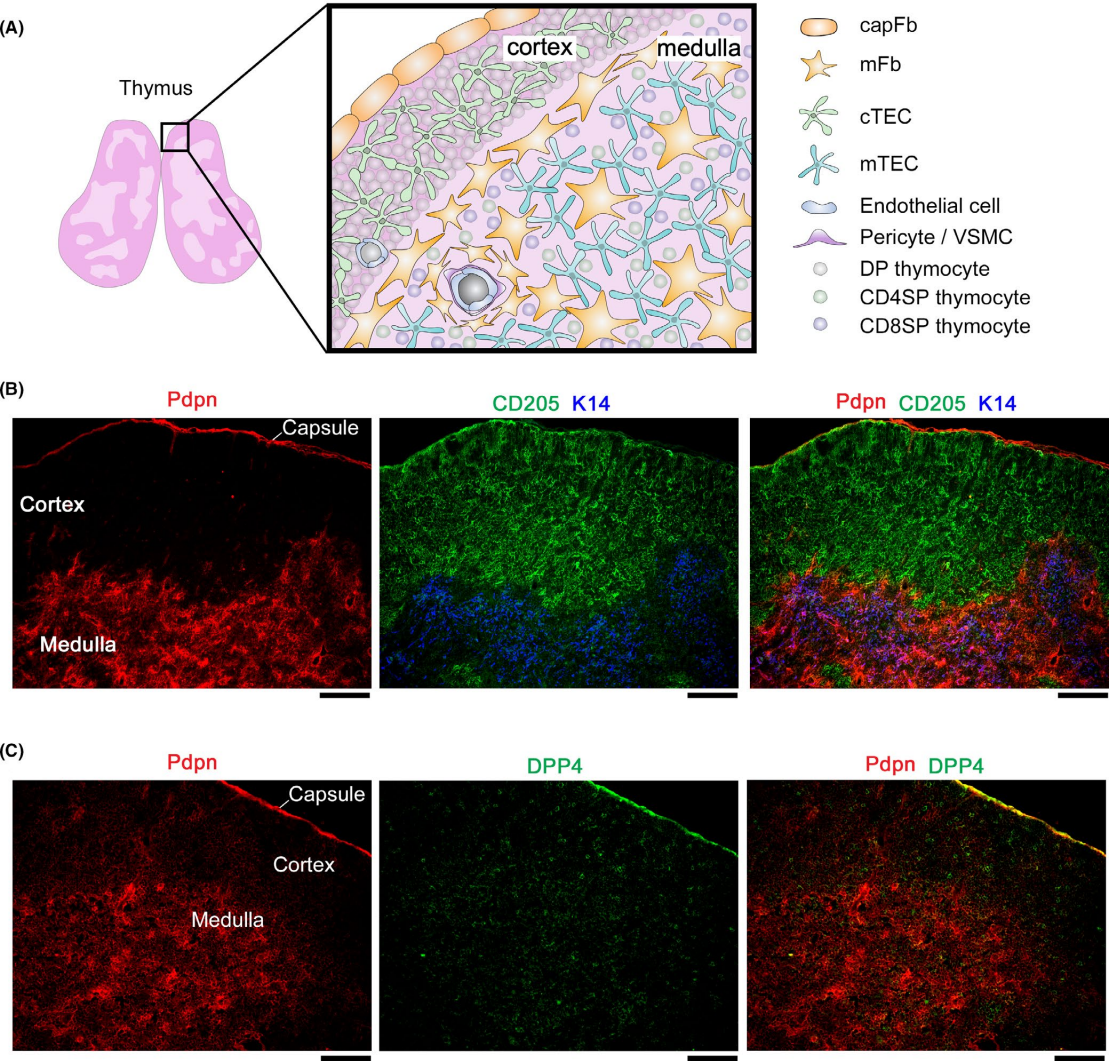
Thymic architecture and stromal cell localization. (A) Schematic depicting the thymic structure and the localization of stromal cells and thymocytes. (B and C) Thymus sections from 5‐week‐old C57BL/6 mice were stained for the indicated markers; Pdpn (fibroblasts), CD205 (cTECs), keratin 14 (K14) (mTECs), and DPP4 (capFbs). The scale bars indicate 100 µm. (B) Thymic fibroblasts expressing Pdpn are localized in the medulla as well as the capsule of the thymus. (C) DPP4 expression segregates the Pdpn^+^ thymic fibroblasts into capFbs and mFbs

The thymus parenchyma is subdivided into two regions, the cortex and medulla, wherein distinct subsets of thymic epithelial cells (TECs) form a reticular meshwork that houses developing thymocytes.^3^, ^4^ The cortex is the outer region with cortical TECs (cTECs) and thymocytes of immature stages, while the medulla is the inner region and is characterized by medullary TECs (mTECs) and mature thymocytes (Figure 1A). TECs play an essential role in T cell development, providing various signals in support of the survival, proliferation, migration, differentiation, and repertoire selection of thymocytes.

Early T‐cell progenitors (ETPs) from the fetal liver or adult bone marrow differentiate into CD4^−^CD8^−^ (double negative, DN) thymocytes in the thymic cortex. Guided by cTECs, DN thymocytes are committed to the T‐cell lineage and undergo rearrangements of the genes encoding the TCR.^3^, ^4^, ^5^ In the adult thymus, ETPs arrive at the cortico‐medullary junction (CMJ) where blood vessels are enriched, and developing DN thymocytes migrate through the cortex toward the subcapsular region and differentiate into CD4^+^CD8^+^ (double positive, DP) thymocytes in the outer cortex. DP thymocytes that have completed gene rearrangement express the rearranged TCR on the cell surface. Upon interaction between the TCR and self‐peptide/MHC complexes, the cells with functional TCR are induced to differentiate into CD4^+^CD8^−^ (CD4 single positive, CD4SP) or CD4^−^CD8^+^ (CD8SP) thymocytes (positive selection), while cells expressing self‐reactive TCR are deleted (negative selection).^6^ The positive selection of such a diverse TCR repertoire depends on the ability of cTECs to produce and present a unique set of self‐peptides via MHC molecules.^7^, ^8^, ^9^, ^10^, ^11^, ^12^

Positively selected SP thymocytes migrate from the cortex to the medulla, attracted by chemokines produced by mTECs.^13^, ^14^, ^15^, ^16^ In the medulla, mTECs express a large number of highly diverse antigens called tissue‐restricted antigens (TRAs) that represent almost all of the tissues in the entire body.^17^, ^18^ The coordination of the mTEC expression of TRAs and relocation of SP thymocytes ensures the negative selection and/or regulatory T cell (Treg) conversion of SP thymocytes that recognize such TRAs, thus establishing self‐tolerance in T cells.^15^, ^16^, ^19^, ^20^, ^21^, ^22^, ^23^, ^24^

In addition to TECs, which control T cell differentiation and selection, a variety of non‐TEC stromal cells support the thymic microenvironment. The blood vasculature is an important parenchymal component of the thymus that supplies oxygen and nutrients and provides entry and exit points for T cells as well as other immune cells. The cortex contains a network of capillaries, while the CMJ and medulla are enriched with arterioles and postcapillary venules.^25^, ^26^ These are made up of functionally distinct endothelial cells that control the influx of bloodborne molecules as well as ETP ingress and mature T cell egress.^27^, ^28^, ^29^

The thymus also contains mesenchymal cells that originate from neural crest (NC) cells. These NC‐derived mesenchymal cells are important for the differentiation and expansion of TECs during embryogenesis. In the postnatal thymus, the mesenchymal cells are predominantly found as fibroblasts in the capsule and medulla, and also as vascular mural cells (Figure 1A). However, despite their abundance in the thymus, the immunological significance of thymic fibroblasts in the postnatal thymus has been both less explored and understood than that of TECs.

Fibroblasts have been generally considered to be ordinary cells without specific features, distributed in tissues throughout the body. However, recent studies have revealed the functional heterogeneity of fibroblasts under various physiological and pathological conditions,^30^, ^31^, ^32^ including immune responses in secondary lymphoid organs^33^, ^34^ or upon viral infection.^35^ This review will provide a current state‐of‐the‐art overview of thymic fibroblasts, focusing on historical studies and recently reported findings on their nature and immunological functions.

## 2 FIBROBLASTS IN THE THYMUS

### 2.1 Overview of thymic mesenchymal cells

In the adult thymus, NC‐derived mesenchymal cells are predominantly found in the capsule and medulla.^36^, ^37^ The capsule of the mouse thymus comprises a monolayer of fibroblasts (capsular fibroblasts, capFbs) that covers the surface of the thymic parenchyma.^25^ The human thymus is covered by a capsule from which interlobular septa arise and divide the parenchyma into lobes.^38^, ^39^ There are some sparsely distributed fibroblasts in the cortex, but their structural features are not presently known. In the medulla, NC‐derived cells are found as medullary fibroblasts (mFbs) and vascular mural cells. mFbs form the reticular network^40^ as well as the blood vessel adventitial layer.^41^ Mural cells are subdivided into pericytes and vascular smooth muscle cells (VSMCs), both of which are embedded in the basement membrane and ensheath the endothelial tubes. Pericytes and VSMCs are distinguished by the absence or the presence of contractility.^42^, ^43^ Although the definition of these cells has been different or even deemed controversial in different studies, in this review we define pericytes as non‐contractile cells and VSMCs as contractile cells expressing α‐smooth muscle actin (α‐SMA).

Traditionally, monoclonal antibodies such as ER‐TR7 and MTS‐15 have been used for the detection of thymic fibroblasts. ER‐TR7 reacts with an unidentified intracellular epitope of fibroblasts,^44^ while MTS‐15 binds glycosphingolipid on the fibroblast surface.^45^, ^46^, ^47^ It was shown that in flow cytometry approximately one‐half of PDGFRα^+^ thymic fibroblasts are MTS‐15^+^.^47^ Although these antibodies recognize not only fibroblasts but also endothelial cells and mural cells, studies using these antibodies have led to the discovery of more specific molecular markers of thymic fibroblasts.

**FIGURE 2:**
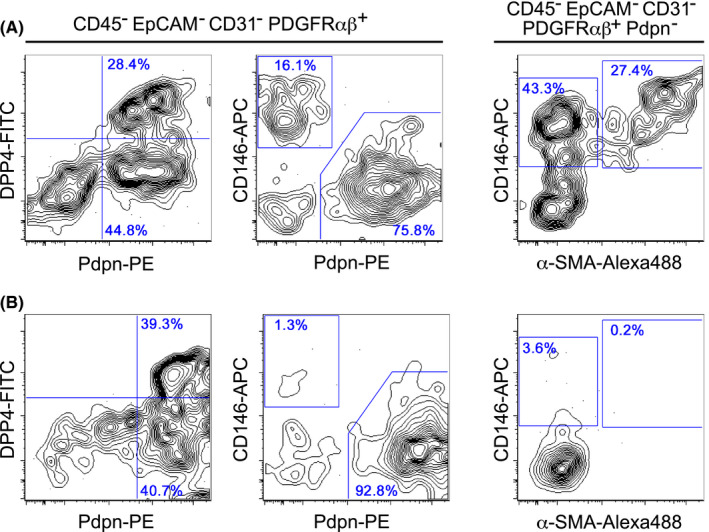
Flow cytometry detection of thymic fibroblast subsets. Thymic stromal cells were prepared using 0.01% Liberase TM (Roche) (A) or 0.125% collagenase D (Roche) (B) from 5‐week‐old C57BL/6 mice, as described previously.^71^, ^72^ Representative flow cytometry profiles of gated stromal cell populations are shown. (A) In the Liberase TM‐dissociated, CD45^−^ EpCAM^−^ CD31^−^ PDGFRαβ^+^ cell population, Pdpn^+^ DPP4^+^ cells (capFbs) and Pdpn^+^ DPP4^−^ cells (mFbs) were detected. The Pdpn^−^ CD146^+^ cells contain α‐SMA^−^ pericytes and α‐SMA^+^ VSMCs. (B) In the collagenase D‐dissociated cell suspension, the Pdpn^−^ CD146^+^ α‐SMA^−^ pericytes and Pdpn^−^ CD146^+^ α‐SMA^+^ VSMCs were found at a very low frequency or were almost undetectable, while capFbs and mFbs were readily detectable

As summarized in Table 1, several proteins have been reported as thymic stromal cell subset markers, including fibroblasts. PDGFRα and PDGFRβ are markers widely used for detecting thymic fibroblasts.^48^, ^49^, ^50^ PDGFRα is highly expressed in capFbs and mFbs, while PDGFRβ is prominent in pericytes and VSMCs. capFbs and mFbs also express podoplanin (Pdpn, also called gp38) and CD34 at high levels.^40^, ^41^ Pericytes and VSMCs can be distinguished from fibroblasts by their high expression of Mcam (CD146) and integrin α7 (Itga7).^41^, ^51^ These markers allow the detection of thymic fibroblast subsets by immunohistochemistry (Figure 1B,C) and flow cytometry (Figure 2), as described below (Section 2.3).

**TABLE 1:** Molecular markers of mouse thymic stromal cells

Protein	Gene	Expression pattern							References
		capFb	mFb	PC	VSMC	EC	cTEC	mTEC	
PDGFRα (CD140a)	*Pdgfra*	++	++	+	+	+/−	−	−	36, 48, 49, 50, 71, 73
PDGFRβ (CD140b)	*Pdgfrb*	+	+	++	++	+/−	−	−	36, 49, 50, 73
Podoplanin (gp38)	*Pdpn*	++	++	−	−	−	+/−	+/−	40, 41, 72, 73, 133
FSP1 (S100A4)	*S100a4*	+	+	++	++	+	−	+	132
CD34	*Cd34*	++	++	‐	−	++	‐	−	41, 73
DPP4 (CD26)	*Dpp4*	++	−	−	−	−	−	−	72
Endosialin (CD248)	*Cd248*	++	+	+	+	−	−	−	97
PECAM‐1 (CD31)	*Pecam1*	−	−	−	−	++	−	−	37, 45, 69, 71
Mcam (CD146)	*Mcam*	−	+/−	++	++	+	−	−	72, 73
Integrin α7	*Itga7*	−	−	++	++	−	−	−	41
α‐SMA	*Acta2*	−	−	−	++	−	−	−	36, 37, 41, 50
Ly51 (CD249)	*Enpep*	−	−	+	+	−	++	−	45, 46, 71
EpCAM (CD326)	*Epcam*	−	−	−	−	−	++	++	45, 46, 71
MHC‐II	*H2‐Aa,H2‐Ab1*	−	−	−	−	−	++	++	45, 70, 71
	*H2‐Ea,H2‐Eb1*								
CD80	*Cd80*	−	−	−	−	−	−	++	71

Note

Abbreviations: capFb, capsular fibroblast; cTEC, cortical thymic epithelial cell; EC, endothelial cell; mFb, medullary fibroblast; mTEC, medullary thymic epithelial cell; PC, pericyte; VSMC, vascular smooth muscle cell.

−, negative; +, positive; ++, strongly positive; +/−, partially positive.

FSP1 and α‐SMA are intracellular proteins, and the others are cell surface proteins.

Most of our understanding of thymic fibroblasts has come from studies using the thymus of animals such as mice and rats. Even though human thymus samples can be obtained from aborted fetuses or neonatal cardiac surgery, these specimens are generally not readily available in many countries. Therefore, previous studies on human thymic fibroblasts have been limited mainly to histological observations using fixed thymus specimens. Recently, however, some have attempted to clarify the functional classification and age‐related changes of human thymic fibroblasts using new technologies such as single‐cell transcriptomics. These studies will be discussed in detail later (Section 2.5).

In order to understand how these thymic fibroblasts develop and are localized within the thymus, it is necessary to have a close look at the organogenesis of the thymus.

### 2.2 Mesenchymal cells in organogenesis of the thymus

The thymus originates from the 3rd pharyngeal pouch, a temporary embryonic structure composed of evaginated endodermal epithelial cells.^52^ The epithelial cells are surrounded by NC‐derived mesenchymal cells, which support pouch patterning, organogenesis of the thymus as well as parathyroid grand, and differentiation of the epithelial cells into TECs. Along with the proliferation of TECs and organization of epithelial parenchyma, the surrounding mesenchymal cells form the capsule that covers the surface, while a fraction of these cells invaginate into the thymus across the epithelial layers to establish an intrathymic network of fibroblasts.^49^, ^53^ Along with this migration, mesoderm‐derived progenitor cells enter into the thymus and differentiate into blood vessel endothelial cells in order to form a vascular network.^54^ Thus, the thymic epithelial, mesenchymal, and endothelial cells spatially and functionally interact in a coordinated manner in order to organize the thymic microenvironment.

NC‐derived mesenchymal cells are required for the differentiation and proliferation of TECs, thus maximizing the thymic capacity for T cell production.^48^, ^49^, ^53^, ^55^, ^56^ The production of extracellular matrixes secreted by mesenchymal cells may be important for incorporating immature TECs into a three‐dimensional microenvironment and presenting cytokines to developing thymocytes.^57^, ^58^ Mesenchymal cell‐derived signaling proteins that control fetal TEC differentiation and expansion have been reported, including insulin‐like growth factor‐1 (IGF1), IGF2, fibroblast growth factor‐7 (FGF7), FGF10, bone morphogenic protein‐4 (BMP4), and the Wnt ligands.^48^, ^56^, ^59^, ^60^, ^61^, ^62^, ^63^, ^64^ Mesenchymal cells produce the vitamin A metabolite retinoic acid, which inhibits TEC proliferation in embryonic thymus.^65^ Thus, thymic mesenchymal cells may also exert a negative regulatory function on TECs.

**FIGURE 3:**
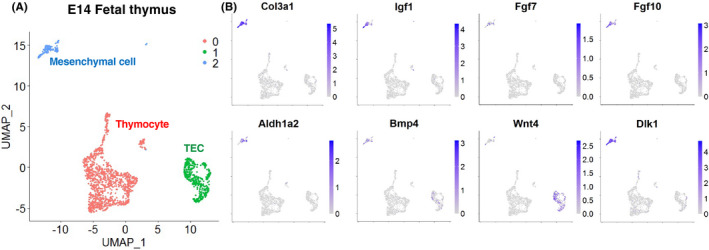
Single‐cell transcriptome of fetal thymic cells. (A) Two‐dimensional representation of E14 fetal thymic cells. Data from the Gene Expression Omnibus (GEO) database under accession no. GSE107910 were used for UMAP clustering.^66^ Each dot represents a single cell. The full source code for analysis is available in GitHub (https://github.com/nittatakeshi/ImmunolRev_Fig3). (B) Expression profiles of the genes which are known to control fetal TEC differentiation and expansion. *Col3a1* was used as a marker of mesenchymal cells

Kernfeld et al performed single‐cell RNA sequencing (RNA‐seq) of whole cell types from embryonic thymus, including mesenchymal cells and TECs.^66^ Figure 3 shows the uniform manifold approximation and projection (UMAP) clustering of their data (GSE107910). *Igf1*, *Fgf7*, *Fgf10*, and *Aldh1a2* (a gene encoding an enzyme for retinoic acid biosynthesis) were specifically expressed in mesenchymal cells (cluster 2), suggesting the non‐redundant role of mesenchymal cells as a source of these key factors. *Bmp4* is expressed in both mesenchymal cells and TECs (cluster 1), consistent with a previous report that the deletion of *Bmp4* in both NC‐derived cells and endoderm‐derived cells (but not either one alone) resulted in defects in thymus organogenesis.^62^ The Wnt ligand *Wnt4* is reported to induce the expression of FoxN1 in TECs^63^ and is highly expressed in TECs, but only slightly in mesenchymal cells, suggesting a role for the Wnt pathway in fetal TEC differentiation, mainly in an autocrine manner. This single‐cell study also revealed that the thymic mesenchyme strongly expresses *Delta‐like non‐canonical Notch ligand 1* (*Dlk1*, also called *Pref1*), which is reported to support thymocyte cellularity in organ culture.^67^

Fetal thymic mesenchymal cells are thought to be a heterogeneous mixture of cells with different characteristics rather than a homogeneous population, and are remotely located in the outer and inner sites of the thymus. However, the mechanism underlying their heterogeneity has yet to be elucidated.

In the following Sections 2.3 to 2.5, recent advances in the identification and characterization of fibroblast subsets as well as other mesenchymal cells in the adult thymus by flow cytometry and transcriptome analyses will be reviewed.

### 2.3 Flow cytometry of the thymic fibroblast subsets

Since several different markers are co‐expressed in different cell types (Table 1), thymic stromal cell subsets have been difficult to evaluate by histological studies alone. An early study by Izon et al applied flow cytometry analysis to characterize thymic stromal cells.^68^ Later studies improved on this method, establishing protocols to efficiently dissociate cells from thymus tissue and to distinguish different stromal cell populations using multicolor cytometry.^45^, ^46^, ^69^, ^70^ To date, the collagenase extracted from *Clostridium histolyticum* has been widely used for dissociating thymic stromal cells. Liberase research grade enzymes, a blend of purified collagenase and other proteases, are able to dissociate thymic epithelial cells in higher yield than using crude collagenase products,^71^ and are now most widely used as the standard protocol. Although they cleave certain cell surface epitopes and thereby weaken the staining intensity, Liberase enzymes are also useful for preparing non‐epithelial thymic stromal cells including fibroblasts, endothelial cells, and vascular mural cells in high yield and quality. Figure 2A shows the flow cytometry profiles of thymic mesenchymal stromal cells (Ter119^−^ CD45^−^ EpCAM^−^ CD31^−^ PDGFRαβ^+^) dissociated from the mouse thymus with Liberase TM. Among the thymic mesenchymal cells, a dominant population is the Pdpn^+^ CD146^−^ fibroblasts, which are characterized by a high expression of PDGFRα and CD34.^41^ A minor population, Pdpn^−^ CD146^+^ cells co‐expressing PDGFRβ, Ly51, and integrin α7,^41^ contains α‐SMA^−^ pericytes and α‐SMA^+^ VSMCs.

Recently, we developed a gradual method of digestion using the Liberase enzyme that allows for the fractionation of thymic cells based on their location within the thymus.^72^ This location‐based fractionation method allows the physical separation of remotely localized thymic fibroblast subsets, capFbs, and mFbs. We identified a cell‐surface protein, dipeptidyl peptidase‐4 (DPP4, also called CD26), which is highly expressed in capFbs but not in mFbs, and consequently established a method to separate capFbs (DPP4^+^ Pdpn^+^) and mFbs (DPP4^−^ Pdpn^+^) by flow cytometry (Figure 2A) as well as histological staining (Figure 1C).

Among the thymic stromal cells, mural cells are relatively difficult to dissociate. When dissociated with collagenase D, a crude collagenase preparation, the yield of Pdpn^−^ CD146^+^ mural cells, including pericytes and VSMCs is very low or even undetectable compared to the yield when Liberase TM is used (Figure 2B). This might be due to the excessive cellular damage caused by contaminating components such as endotoxin in the crude enzyme preparations, and possibly explains the reason why these cells have not been readily detected in previous studies (see Section 2.5). Even with Liberase, it is still possible that some unnoticed stromal cell types are lost during enzymatic digestion.

Collectively, however, by using cell dissociation with Liberase and multicolor flow cytometry, it has now been made possible to determine and isolate almost all of the types of stromal cells that compose thymic microenvironment.

### 2.4 Population‐based transcriptome profiling of thymic fibroblasts

In order to characterize the nature and function of thymic fibroblasts, many studies have sought to reveal their gene expression profiles. However, it has been difficult to delineate a unified gene expression pattern for thymic fibroblasts, because the markers used to isolate the cell subsets vary from study to study.

Patenaude and Perreault performed whole transcriptome analysis by RNA‐seq of thymic mesenchymal cells (Lineage^−^ EpCAM^−^ CD31^−^ Sca1^+^).^73^ Their results revealed that Sca1^+^ thymic mesenchymal cells exhibit a higher expression of genes involved in epithelial interaction, apoptotic cell clearance, and T‐cell progenitor expansion, compared to their bone or skin counterparts, suggesting a pivotal role for these cells in the thymic microenvironment. However, since the Sca1^+^ mesenchymal cell population is a mixture of the fibroblasts, pericytes, and VSMCs, the cell subsets which express each of the key genes remain to be determined.

In a study by Sitnik et al, thymic mesenchymal cells (Lineage^−^ EpCAM^−^ CD31^−^ PDGFRβ^+^) were divided into two subsets, Pdpn^+^ Ly51^−^ fibroblasts and Pdpn^−^ Ly51^+^ mural cells, and then, their entire transcriptome was analyzed by microarray.^41^ The Pdpn^+^ Ly51^−^ cells expressed genes regulating vascular and epithelial cell growth (*Vegfc*, *Vegfd*, *Igf1*, *Igf2*, *Fgf2*, *Fgf7*, and *Fgf10*), suggesting that thymic fibroblasts play a role in maintaining vascular and epithelial niches. It is likely that the Pdpn^−^ Ly51^+^ cells contained VSMCs, as indicated by the high expression of the *Acta2* gene (α‐SMA).

Our recent results have determined the whole transcriptome of the isolated thymic fibroblast subsets by RNA‐seq.^72^ Both capFbs and mFbs highly express certain fibroblast‐associated genes such as collagens (*Col1a1*, *Col1a2*, *Col3a1*, and *Col6a1*), extracellular matrix proteins (*Dcn*, *Lum*, *Mgp*, and *Sparc*), extracellular proteases (*Htra1*, *Htra3*, *Mmp2*, *Mmp3*, and *Mmp14*), and protease inhibitors (*Serping1* and *Serpinh1*). These gene expression signatures are similar to those of fibroblastic cells in secondary lymphoid organs reported in previous studies.^41^, ^73^ A set of genes was found to be differentially expressed in capFbs and mFbs (see Sections 3 and 4).

We also analyzed the transcriptome of Pdpn^−^ CD146^+^ mural cells. These cells displayed a gene expression pattern reminiscent of a certain type of fibroblast (*Col3a1*, *Col4a1*, and *Col6a1*) and a potent expression of genes associated with pericytes (*Cspg4*) or muscle cells (*Acta2*, *Myl9*, and *Myh11*), indicating that the Pdpn^−^ CD146^+^ cell population is a mixture of pericytes and VSMCs. This is consistent with the results of flow cytometry that indicate Pdpn^−^ CD146^+^ cells comprise α‐SMA^−^ pericytes and α‐SMA^+^ VSMCs (Figure 2A).

These population‐based transcriptome datasets of thymic fibroblast subsets should provide a powerful tool for understanding the development and function of the thymic microenvironment, especially in combination with the recently advanced single cell–based transcriptome datasets described below.

### 2.5 Single cell–based transcriptome profiling of thymic fibroblasts

**FIGURE 4:**
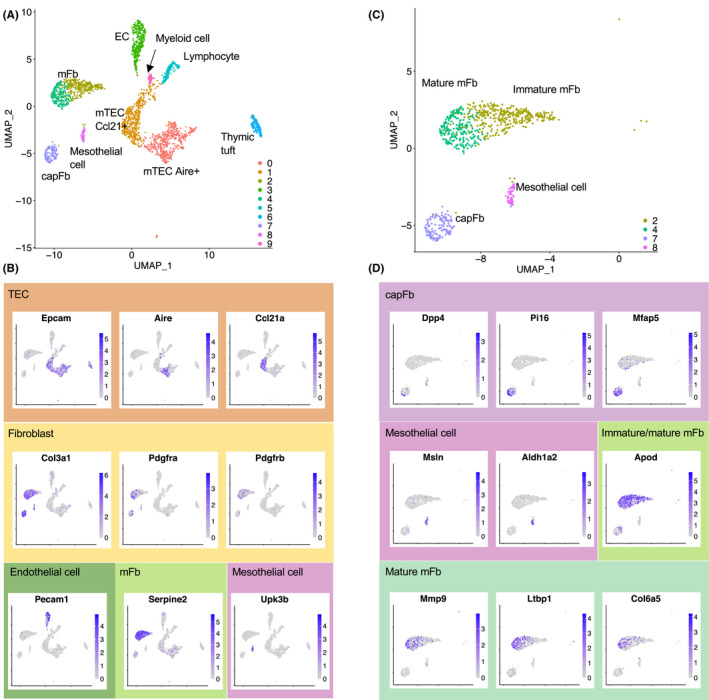
Single‐cell transcriptome of adult thymic stromal cells. Single‐cell RNA‐seq data of mouse thymic stromal cells (GEO accession no. GSE103967, Experiment ID thymus_stroma_WT) were used for UMAP clustering.^74^ The full source code for analysis is available in GitHub (https://github.com/nittatakeshi/ImmunolRev_Fig4). (A) Two‐dimensional representation of cells by UMAP. Each dot represents one cell. (B) Projection of representative genes. Clusters 0, 1, and 6 are TEC subsets defined by the expression of *Epcam* as well as key genes such as *Aire* or *Ccl21a*. Cluster 3 represents endothelial cells (ECs) expressing *Pecam1*. Clusters 2, 4, 7, and 8 represent thymic fibroblasts characterized by the expression of *Col3a1*, *Pdgfra*, and *Pdgfrb*. Contaminating lymphocytes (cluster 5) and myeloid cells (cluster 9) are also included. (C) Thymic fibroblast subsets (clusters 2, 4, 7, and 8) are highlighted. (D) Cluster 7 represents capFbs expressing *Dpp4*, *Pi16*, and *Mfap5*. Cluster 8 represents mesothelial cells defined by a high expression of *Msln*. mFbs (clusters 2 and 4) can be subdivided into immature and mature mFbs, and the latter express *Mmp9*, *Ltbp1*, and *Col6a5*

Bornstein et al reported single‐cell RNA‐seq analysis of mouse thymic stromal cells.^74^ Figure 4 shows the UMAP clustering of their data of whole thymic stromal cells (GSE103967). The TEC subpopulations, endothelial cells, and contaminating lymphocytes and myeloid cells were clustered according to the gene expression signatures specific for each cell types (Figure 4A,B). Clusters 2, 4, 7, and 8 exhibited a high level of expression of *Col3a1*, *Pdgfra*, and *Pdgfrb*, indicating that these clusters comprise thymic fibroblasts. Cluster 7 expressed *Dpp4*, *Pi16*, and *Mfap5*, corresponding to capFb (Figure 4C,D) based on the results of the population‐based transcriptome.^72^ Both clusters 2 and 4 exhibited a high expression of *Serpine2* and *Apod* and no expression of *Dpp4*, corresponding to mFb. Pericytes and VSMCs were not clearly clustered at this resolution, most likely because most of these cells were lost during the collagenase D digestion performed for cell isolation.^74^

Cluster 8 is a relatively minor cell population that expresses the *Dpp4* and *Pdpn* that are similar to cluster 7 (capFb), but also exhibits a high expression of *Msln* and *Upk3b* with only negligible expression of *Pi16* and *Mfap5*, which is markedly different from cluster 7. It is likely that cluster 8 comprises mesothelial cells that can be defined by a high expression of mesothelin (*Msln*).^75^, ^76^ These mesothelial cells are specifically detectable in the outermost fractions of the thymus (unpublished data), suggesting that these cells compose the thymic capsule together with capFbs, or that they are derived from the visceral pleura in contact with the thymus. Thus, the DPP4^+^ Pdpn^+^ cells that we called capFbs (Section 2.2 and Figure 2A) contain two subpopulations, a major population expressing *Pi16* and a minor population corresponding to mesothelial cells. In this review, we refer to the former as capFb.

In a recent study, Park et al performed single‐cell RNA‐seq to create a comprehensive atlas of human thymic cells including stromal cells.^77^ In their dataset, thymic mesenchymal cells were classified into fibroblast type 1 (Fb1), fibroblast type 2 (Fb2), cycling fibroblasts, and VSMCs.^78^ Fb1 and Fb2, respectively, correspond to mesothelial cells and capFbs in the mouse (Figure 4C,D). These clustering results did not contain clusters that correspond to mouse mFbs, probably due to the large variability in human data, which includes a variety of samples in a range from the fetus to the adult.

The same group also published a single‐cell RNA‐seq dataset of the mouse thymus^78^ generated with previously reported data from the fetal and postnatal thymus,^66^, ^74^ in which thymic mesenchymal cells were classified into four groups, Fb_Aldh1a2 (corresponding to mesothelial cells), Fb_Pi16 (corresponding to capFb), Fb_Postn (corresponding to mFb), and VSMCs (containing pericytes and VSMCs). These clusters are all consistent with the cell populations revealed by flow cytometry and the population‐based transcriptome.

In the following sections, we will focus on the thymic fibroblast subsets capFb and mFb, summarizing how they develop and regulate T cell development, referring to the studies with transcriptome data as well as genetically modified mouse models.

## 3 THE CAPSULAR FIBROBLAST (CAPFB)

### 3.1 Development of capFbs

The surface of the thymus is covered by a monolayer of fibroblasts that contacts the epithelial parenchyma across the basement membrane. capFbs are derived from the NC‐derived mesenchymal cells that surround the embryonic thymus primordium and remain outside. In the mouse, at around E13, thymic mesenchymal cells diverge into two populations, a perithymic cell population remaining outside the organ that forms the thymic capsule, and another population that migrates into the thymus across the epithelial layers to give rise to mFbs, pericytes, and VSMCs. At E15, DPP4 begins to be expressed in the capsular populations, which allows the two populations capFb and mFb to be distinguished by flow cytometry analysis.^72^ It was also shown that in human thymus the fibroblasts expressing *DPP4* and *PI16* (likely capFbs) increase during fetal development.^77^ The mechanisms that induce the expression of capFb‐associated genes, including *Dpp*4, are still unclear.

DPP4 is a useful marker for the detection and isolation of capFbs. DPP4 is reportedly expressed by activated fibroblasts in fibrotic tissues such as the skin of patients with systemic sclerosis as well as in cases of liver fibrosis^79^, ^80^, ^81^, ^82^ or the breast implant capsule in patients with capsular contraction.^83^, ^84^ Genetic ablation or pharmacological inhibition of DPP4 ameliorates fibrosis in mice, indicating that DPP4 activity is important for fibroblast activation and tissue fibrosis.^82^, ^85^ Whether DPP4 is involved in the function of the thymic capsule remains to be elucidated.

### 3.2 Control of TEC development by capFbs

**FIGURE 5:**
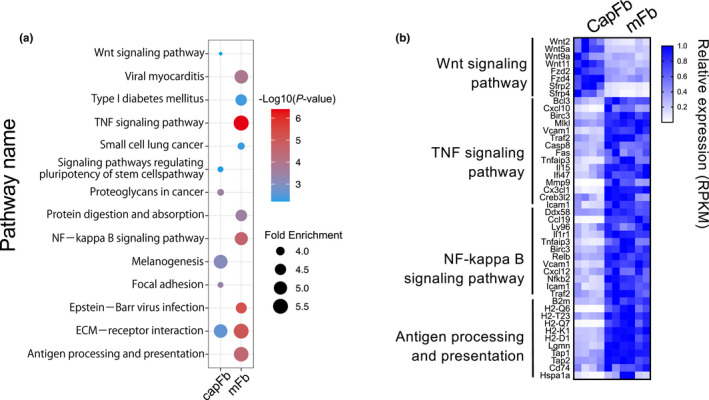
Gene expression profiling of capFbs and mFbs by whole transcriptome analysis. (A) KEGG pathway analysis of genes differentially expressed in capFbs and mFbs. Genes highly expressed in capFbs (capFbs/mFbs > 2, mean RPKM of capFbs > 10, *P* <.05) or in mFbs (mFb/capFb > 2, mean RPKM of mFb > 10, *P* <.05) were collected from bulk RNA‐seq data (GEO accession no. GSE147357)^72^ and subjected to KEGG pathway enrichment analysis using DAVID 6.8. (B) Heat map showing the relative expression of genes categorized as being in the Wnt signaling pathway, TNF signaling pathway, and NF‐κB signaling pathway, as well as antigen processing and presentation

Figure 5A shows the KEGG pathway enrichment analysis of the transcriptome data of the thymic fibroblast subsets.^72^ The Wnt signaling pathway was found to be significantly enriched in capFbs compared with mFbs. Indeed, capFbs express many Wnt family ligands and regulators (*Wnt2*, *Wnt5a*, *Wnt9a*, *Wnt11*, *Sfrp2*, and *Sfrp4*) at higher levels than mFbs as well as other thymic stromal cells (Figure 5B). Wnt signals reportedly critically control the differentiation of TECs and thymocytes,^63^, ^86^, ^87^, ^88^, ^89^ suggesting a role for capFb‐derived Wnt signals in the thymus. Although a previous study demonstrated that TECs themselves act as a source of Wnt ligands for maintaining TEC cellularity and thymus size,^90^ the contribution of capFb‐derived Wnt signals to the regulation of TEC and T cell development remains to be determined in future study.

Thymic mesenchymal cells (Pdpn^+^ Ly51^−^) are a major thymic source of retinoic acid, which exerts an inhibitory effect on TEC proliferation.^65^ The transcriptome data show that the genes encoding retinoic acid‐producing enzymes *Aldh1a1*, *Aldh1a2*, and *Aldh1a3* are strongly expressed in capFbs and/or mesothelial cells: *Aldh1a1* in both, *Aldh1a2* in mesothelial cells, and *Aldh1a3* in capFbs (Figure 4D and data not shown). Mice with TECs unable to respond to retinoic acid display an aberrant cTEC phenotype, including increased proliferation and the accumulation of an immature population, with a subsequent reduction in thymic cellularity.^91^ Thus, the retinoic acid produced in the outermost layer of the thymus acts as a regulator of TECs and is important for normal T cell development.

### 3.3 Control of T cell development by capFbs

The genes uniquely expressed in capFbs in the thymus include the extracellular protease DPP4 (*Dpp4*), peptidase inhibitor‐16 (*Pi16*), Wnt ligands, and semaphorin ligands (*Sema3c* and *Sema3d*). The semaphorin ligands expressed in capFbs may control the migration of developing thymocytes, since it is reported that the semaphorin receptor Plexin D1 is strongly expressed by DP thymocytes and the absence of Plexin D1 disturbs the medulla localization of newly generated SP thymocytes.^92^

CD248 (Endosialin) is known to be a marker of mesenchymal cells.^93^, ^94^, ^95^ Immunohistochemical analysis indicated that CD248 expression is prominently detected in the perithymic mesenchyme in the mouse embryo, then downregulated postnatally.^96^, ^97^ From transcriptome analysis of human and mouse thymus, *Cd248* mRNA is expressed at the highest level in capFbs and mesothelial cells,^72^, ^77^, ^78^ suggesting that CD248 may exert effects in the outermost niches of the thymus. CD248‐deficient mice display age‐dependent decline of thymus size and thymocyte cellularity, and, in particular, a marked reduction of DN3 thymocytes.^97^ The proliferation of DN3 thymocytes that occurs in the subcapsular zone may be regulated by the CD248 expressed in capFbs. It was also shown that CD248‐deficient mice exhibit delayed recovery of thymus size and vascularization following infection‐induced atrophy. Although the mechanism remains unclear, CD248 may promote re‐vascularization and thymocyte growth during postinfection regeneration.

How capFbs and mesothelial cells control the outermost barrier of the thymus and thymus integrity needs to be clarified in future. The interplay between capFbs and subcapsular cTECs may also be important for supporting T cell development in the subcapsular zone and outer cortex, but determining its physiological significance and molecular basis still remains a challenge.

## 4 THE MEDULLARY FIBROBLAST (MFB)

### 4.1 Development of mFbs

On histological analysis, mFbs are detected as a reticular structure interwoven with but also clearly separated from the network of mTECs.^40^, ^72^ A population of mFbs expressing CD34 forms adventitial layers that surround mural cells and endothelial cells, and thus referred to as adventitial cells.^41^ In flow cytometry analysis combined with the location‐based fractionation method, mFbs are found to be enriched in the medullary fraction together with mTECs, consistent with the histological findings.^72^

Studies using a fate‐mapping strategy with various Cre lines specific to NC (Wnt1a‐Cre, Sox10‐Cre, or Twist2‐Cre) or thymic epithelium (Foxn1‐Cre) have demonstrated that mFbs are derived from the NC‐derived mesenchymal cells that surround the embryonic thymus primordium, not from TECs^36^, ^37^, ^72^ (this is also discussed in Section 5). mFbs in the fetal thymus express low levels of marker proteins such as Pdpn and ICAM1, and their expression increases in the course of postnatal development,^41^ indicating that the maturation of mFbs is developmentally regulated during ontogeny (see Section 4.3). It has also been shown that Pdpn^hi^ ICAM1^hi^ mFbs are capable of generating Pdpn^−^ Esam1^+^ mural cells or lymph node stroma‐like cells when reaggregated under appropriate conditions.^41^ Hence, the mFbs at the population level contain progenitor cells for multiple lymphoid tissue stromal cells, and their differentiation capacity is dependent on the particular environmental context.

### 4.2 Gene expression in mFbs

As a result of KEGG pathway analysis, the genes for TNF signaling and NF‐κB signaling as well as antigen processing and presentation were found to be significantly enriched in mFbs compared with capFbs (Figure 5A). These findings suggest that TNF signaling and NF‐κB activation pathways play important roles in development and/or function of mFbs and that mFbs are differentiated such that they have a higher antigen presentation capacity than capFbs. Also, a set of genes, including certain collagens (*Col6a5*, *Col6a6*), matrix metalloprotease‐9 (*Mmp9*), metabolic enzymes (*Hmgcs2*, *Ltc4s*, and *Qprt*), and TGFβ‐binding proteins (*Ltbp1* and *Ltbp2*) are predominantly expressed in mFbs among all of the thymic stromal cell types.

**FIGURE 6:**
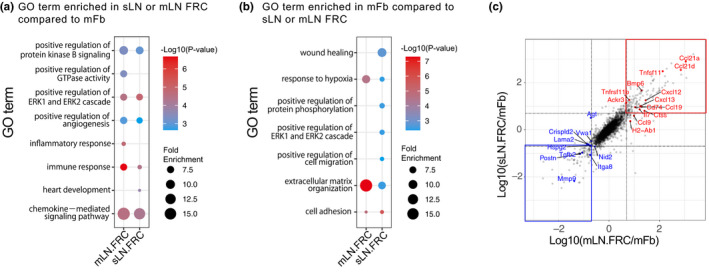
Comparison of the whole transcriptome between thymic mFbs and lymph node FRCs. RNA‐seq data of mFbs, mesenteric lymph node (mLN) FRCs, and skin‐draining lymph node (sLN) FRCs (GEO accession no. GSE147357) were used.^72^ (A, B) GO term enrichment analysis of genes preferentially expressed in lymph node (LN) FRCs or mFbs. Genes preferentially expressed in LN FRCs compared to mFbs (mLN FRCs: mLN FRCs/mFbs > 5, sLN FRCs: sLN FRCs/mFbs > 5) (A) or in mFbs compared to LN FRCs (mLN.FRCs: mFbs /mLN FRCs > 5, sLN.FRCs: sLN FRCs/mFbs > 5) (RPKM > 10 in any of the groups) were subjected to GO term enrichment analysis using DAVID 6.8. (C) Scatter plot of the gene expression ratio between mLN FRCs/mFbs and sLN FRCs/mFbs. The genes associated with the immune response and the chemokine‐mediated signaling pathway in (A) are highlighted in red. The genes associated with extracellular matrix organization in (B) are highlighted in blue

mFbs form a conduit‐like structure that resembles the one formed by fibroblastic reticular cells (FRCs) in the lymph nodes.^40^, ^98^ To determine the functional cue for mFbs, the transcriptome was compared between mFbs and lymph node FRCs.^72^ Gene ontology (GO) term analysis revealed that mFbs are significantly enriched for genes associated with extracellular matrix organization and cell adhesion but have a lower association with genes for angiogenesis, the inflammatory response, and the immune response, suggesting functional differences between these two morphologically similar cell types (Figure 6A,B).

Lymph node FRCs can be divided into several subtypes expressing different sets of key cytokines and chemokines, such as T‐cell zone reticular cells (TRCs) expressing *Ccl19*, *Ccl21a* and *Il7*, follicular dendritic cells (FDCs) expressing *Cxcl13*, marginal reticular cells (MRCs) expressing *Tnfsf11*, and medullary reticular cells (medRCs) expressing *Cxcl12*, *Il6*, *Tnfsf13*, and *Tnfsf13b*.^33^, ^34^, ^99^ Most of these FRC‐associated cytokines and chemokines are not or only just barely expressed in mFbs (Figure 6C),^72^ but are predominantly expressed in TECs or thymocytes,^100^, ^101^, ^102^ suggesting that the roles played by FRCs in the lymph nodes are replaced by TECs and thymocytes in the thymus. mFbs highly express other sets of chemokine genes, such as *Cx3cl1* and *Cxcl14*, that are barely expressed in lymph node FRCs, possibly contributing to the regulation of cell migration in the thymic medulla.^72^ Thus, mFbs comprise a thymic‐specific subset of fibroblasts that is functionally distinct from lymph node FRCs.

It has been reported that patients with autoimmune diseases such as myasthenia gravis or autoimmune‐prone mice exhibit an abnormal accumulation of B cells in the thymus.^103^, ^104^, ^105^ In lupus‐prone BWF1 mice, thymic B cells proliferate within the perivascular space and cluster in structures that resemble ectopic germinal centers, where B cells differentiate to secrete autoantibodies.^106^ Other early studies also reported that the thymus from lupus‐prone NZB mice or diabetes‐prone NOD mice contained giant perivascular spaces, which are filled with mature T cells, B cells, and fibroblast‐associated extracellular matrix proteins.^107^, ^108^, ^109^, ^110^ Whether these autoimmune‐associated, “germinal center‐like” structures involve any subset(s) of thymic fibroblasts, like as the formation of the canonical germinal centers in the lymph nodes requires FDCs, remains an open and interesting question.

### 4.3 LTβR‐dependent maturation of mFbs

Single‐cell RNA‐seq analysis of mouse thymic stromal cells demonstrated that the genes highly expressed in mFbs, such as *Serpine2* and *Apod*, are prominently detected in clusters 2 and 4 (Figure 4C,D). Certain mFb‐associated genes, including *Mmp9*, *Ltbp1*, and *Col6a5*, are detectable in cluster 4 but not cluster 2. These cluster 4‐specific genes are expressed in mFbs from adult but not neonatal mice,^72^ suggesting that clusters 4 and 2 represent mature and immature mFbs, respectively. In addition, most of these mature mFb‐associated genes are expressed under the control of the lymphotoxin signal.

The TNF superfamily ligand lymphotoxin (LTα_1_β_2_) is predominantly expressed by developing SP thymocytes in the thymus and binds to the lymphotoxin β receptor (LTβR) expressed in thymic stromal cells to induce intracellular signal transduction. The LTβR is expressed at the highest level in mFbs among the thymic stromal cells.^72^ In mFbs from LTβR‐deficient mice, the expression of a large fraction of mFb‐associated genes was diminished. Indeed, LTβR‐deficient mFbs displayed a reduced expression of mFb‐associated proteins such as Pdpn, ICAM‐1, and VCAM‐1.^41^, ^111^ Thus, the LTβR signal critically controls the functional maturation of mFbs. It is known that the LTβR signal is required for the maturation of lymph node FRCs,^112^ offering an analogy that shows that these distinct fibroblast subsets share common signaling pathways for maturation.

### 4.4 Self‐antigen expression by mFbs for the induction of immune tolerance

Early studies showed that fibroblasts are capable of presenting self‐antigens to induce the positive selection of thymocytes, suggesting that the ability to mediate positive selection is not limited to the thymic epithelium.^113^, ^114^ However, a subsequent series of studies revealed that positive selection requires proteasomes and lysosomal proteases that are uniquely expressed in cTECs.^7^, ^8^, ^9^, ^10^, ^11^, ^115^, ^116^, ^117^ Also, the major stromal cells that interact with preselected DP thymocytes are cTECs,^118^ and fibroblasts are scarce in the thymic cortex. Therefore, it has been thought that fibroblasts are not important for positive selection. On the other hand, mFbs are localized in the medulla, where the negative selection of TRA‐reactive thymocytes occurs. Transcriptome analysis indicates that mFbs are highly associated with genes for antigen presentation, suggesting a contribution to negative selection (Figure 5A).

**FIGURE 7:**
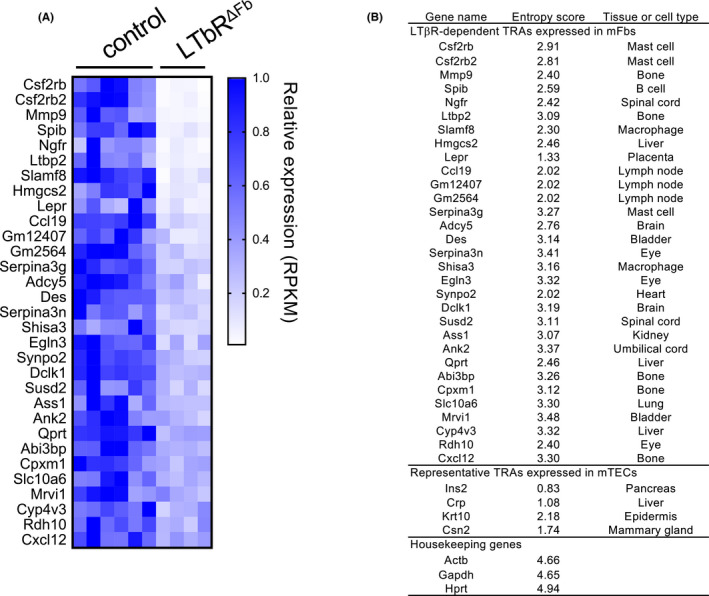
LTβR‐dependent genes in mFbs include TRAs. To define the TRAs, the Shannon entropy score was calculated using the gene expression profiles (GEO accession no. GSE10246).^147^ The full source code for analysis is available in GitHub (https://github.com/nittatakeshi/ImmunolRev_Fig7). Genes with an entropy score of less than 3.5 are defined as TRA genes. Although these TRA genes were extracted from comprehensive transcriptome data by unbiased mathematical calculation, they may also contain genes that encode functional proteins in mFbs and fibroblast lineage‐specific proteins. (A) The gene expression data on the mFbs from Ltbr^ΔFb^ mice (Twist2‐Cre Ltbr^flox/flox^ (n = 4)) compared to those from control mice (C57BL/6 (n = 2) and Ltbr^flox/flox^ (n = 4)) are from an RNA‐seq dataset (GEO accession no. GSE147357).^72^ TRA genes with a mean RPKM >10 in the control mFbs and the ratio of RPKM (Ltbr^ΔFb^ /control) > 0.5 and significant (*P* <.05) are shown. (B) LTβR‐dependent TRA genes expressed in mFbs, representative TRA genes expressed in mTECs, and representative housekeeping genes are listed. Expression specificity was determined by computationally extracting the tissues or cell types that showed the highest mRNA expression values

**FIGURE 8:**
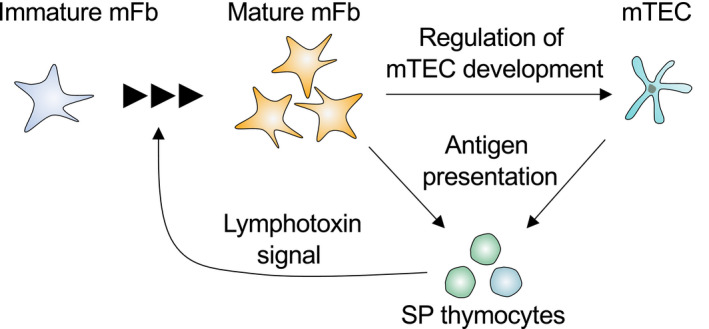
Induction of T cell tolerance by mFbs and mTECs. The immature mFbs give rise to mature mFbs upon interaction with SP thymocytes expressing lymphotoxin (LTα_1_β_2_). The mature mFbs promote mTEC development. Both mTECs and mFbs express and present self‐antigens, thus contributing to the deletion of self‐reactive SP thymocytes and the establishment of central tolerance

Insights into the roles of thymic fibroblasts in T cell selection have come from studies of the LTβR. Pioneering studies by Boehm and colleagues reported that the LTβR expressed in thymic stroma is important for the induction of T cell tolerance,^119^, ^120^ and later studies demonstrated the requirement of LTβR in optimum mTEC differentiation and gene expression.^121^, ^122^, ^123^, ^124^ However, mice lacking LTβR specifically in TECs do not exhibit signs of autoimmunity, while mice systemically lacking LTβR do, indicating that the key target of lymphotoxin signaling in the context of tolerance induction must be non‐TEC stromal cells.^123^, ^124^ Recently, we found that fibroblast‐specific LTβR‐deficient mice displayed signs of autoimmunity against peripheral tissues, similar to systemic LTβR‐deficient mice.^72^ TCR repertoire analysis revealed that certain TCR clones escape negative selection in fibroblast‐specific LTβR‐deficient mice. The LTβR in mFbs controls the expression of a set of mFb‐specific genes. Such LTβR‐dependent genes expressed in mFbs include certain TRAs that have been defined based on mathematical methods for evaluating tissue‐specific gene expression (Figure 7). Mice specifically lacking the LTβR in fibroblasts exhibit a marked production of autoantibodies against these TRAs. Collectively, these findings indicate that mFbs act as a primary source of certain self‐antigens for the induction of T cell tolerance, and the lymphotoxin signal is a key mediator of this process (Figure 8).

The expression of TRAs in mFbs is induced by the non‐canonical NF‐κB pathway downstream of the LTβR.^72^ This is apparently different from the regulatory mechanisms for TRA expression in mTECs, where hundreds to thousands TRAs are expressed by virtue of chromatin modification and transcription regulators such as Aire and Fezf2.^18^, ^19^, ^125^ Considering the large number of self‐antigens encoded by genomes, the induction of central tolerance likely does not rely only on the mTEC expression of TRAs, but instead is achieved by expression in the thymic medulla of genes representing cell types that exist across multiple tissues and organs. The number of TRAs expressed in mFbs may be less than that of mTEC‐expressed TRAs, but mFbs express a set of fibroblast‐specific antigens for developing T cells to delete self‐reactive clones and thereby induce self‐tolerance. It is likely that the thymic medulla needs to be populated with various cell lineages to ensure T cell tolerance to self‐antigens, as indeed mTECs also express antigens specific for various cell lineages by differentiating themselves into peripheral epithelial cells such as keratinocyte‐like cells or tuft cells.^74^, ^126^ These findings have led us to propose that the thymic medulla needs to contain different cell types, each of which express cell type‐restricted antigens (CRAs) to maximize the variety of self‐antigens available for T cell selection.^72^

The expression and presentation of self‐antigens have also been observed in fibroblastic stromal cells in the lymph nodes.^127^ Lymph node stromal cells present antigens to T cells directly, or indirectly through interaction with dendritic cells (DCs).^128^, ^129^ Similarly, in the thymus, mFb‐specific antigens might also be transferred to and presented by thymic DCs so as to induce T cell tolerance, since a substantial portion (about half) of mTEC‐derived self‐antigens are indirectly presented by thymic DCs.^130^, ^131^ Indeed, it was demonstrated that the cytoplasmic proteins produced in thymic fibroblasts can be transferred to thymic DCs.^72^ This mechanism explains how LTβR deficiency in fibroblasts results in the production of autoantibodies against mFb‐specific antigens.

### 4.5 Regulation of mTEC development by mFbs

It is also possible that mFbs indirectly promote T cell tolerance by controlling mTECs, since the fibroblast‐specific deletion of the LTβR causes a reduction in the number of mTECs.^72^ Consistent with this, it was shown that the LTβR signal influences the localization of mFbs and their interaction with mTECs.^50^ In contrast, the loss of mTECs has no influence on mFb cellularity, indicating that mFbs lie upstream of mTECs in the hierarchy of stromal interactions within the medullary microenvironment.^72^ LTβR‐dependent genes such as cell adhesion molecules (ICAM‐1 and VCAM‐1), extracellular proteases (MMP9), and extracellular matrix proteins (collagens and related proteins) might play key roles in controlling the development and/or maintenance of mTECs.

LTβR‐independent mechanisms in fibroblasts also reportedly induce TEC differentiation. IGF1 is predominantly produced by capFbs and mFbs in the thymus, and the administration of IGF1 by continuous infusion induces an expansion of both cTECs and mTECs,^60^ although the physiological significance of this induction has not been clarified by loss‐of‐function studies. FGF7 and FGF10, both reported to be involved in TEC proliferation during embryogenesis, are also expressed in adult thymic fibroblasts. FGF7 is expressed in capFbs, while FGF10 is expressed in mFbs, suggesting different roles for these factors in the regulation of postnatal TECs.

Sun et al reported the role played by fibroblast‐specific protein 1 (FSP1, also called S100a4).^132^ FSP1 is a cytoplasmic and secreted protein expressed in mFbs at higher levels than in capFbs. FSP1 promoter‐driven, inducible cell ablation in mice resulted in a marked reduction of mTECs. Furthermore, FSP1 itself may function as a direct regulator of mTECs, as FSP1‐deficient mice exhibited a smaller sized thymus and reduced number of mTECs, and the addition of purified FSP1 protein increased the mTEC number in organ culture. Collectively, this study demonstrates the pivotal role of FSP1‐expressing fibroblasts in controlling the mTEC number. However, as FSP1 expression (at least at the mRNA level) is not limited to fibroblasts but is also detectable in other thymic stromal cells, including mural cells and mTECs themselves,^72^ so whether FSP1 is a molecule representative of the function of thymic fibroblasts is in need of further clarification. More specific markers or reporters as well as cell ablation systems need to be developed to better investigate the physiological functions of the thymic fibroblast subsets.

### 4.6 Regulation of T cell migration by mFbs

The LTβR signal induces the expression of Pdpn in mFbs. Pdpn is a mucin type glycoprotein expressed in various types of stromal cells and in particular is highly expressed in FRCs in the lymph nodes. In the thymus, Pdpn is expressed in capFbs and mFbs as well as a fraction of TECs (Table 1).^133^, ^134^, ^135^ The extracellular domain of Pdpn binds to various proteins that are secreted by or displayed on other cells.^136^ Pdpn^+^ mFbs form conduit‐like structures in the medulla and bind the chemokine CCL21 produced by mTECs.^40^ In the absence of Pdpn, CCL21 fails to efficiently localize in the medulla, a failure which is accompanied by both inefficient migration and generation of Tregs in the medulla. A similar phenotype is observed in mice lacking CCL21 or CCR7, the receptor for CCL21, suggesting a role for Pdpn‐immobilized CCL21 on mFbs in thymic Treg generation.

Two very recent reports demonstrated that CCL21 is displayed on the surface of mFbs and pericytes around blood vessels.^137^, ^138^ Cell‐surface binding of CCL21 is mediated by the heparan sulfate strongly expressed by these cells, and consistent with this, the EXT family genes, *Ext1* and *Ext2*, that encode the glycosyl transferases for heparan sulfate biosynthesis are highly expressed in mFbs and pericytes.^138^ James et al reported that CCL21 captured by the blood‐vessel surrounding fibroblasts and pericytes promotes T cell emigration from the neonatal mouse thymus, suggesting a synergy between TEC‐dependent production and mesenchymal cell‐dependent immobilization of chemokines for controlling T cell migration.^137^ A study by Hsu et al demonstrated that elimination of heparan sulfate in the thymus by genetic deletion of *Ext1* resulted in a marked decrease in the number of thymic fibroblasts and TECs as well as thymocytes.^138^ Thus, heparan sulfate produced by mesenchymal cells may also be important for thymic stromal cell homeostasis via the immobilization of secreted proteins including chemokines, although the mechanism has not yet been elucidated.

As shown by transcriptome analyses, mFbs themselves express certain chemokine genes, such as *Cxcl14* and *Cx3cl1*, but not *Ccl21*.^72^ Whether and how these mFb‐specific chemokines contribute to cell migration in the thymus and exert immunological functions are still presently unknown.

## 5 FIBROBLASTS IN AGE‐RELATED THYMIC INVOLUTION AND ADIPOSIS

The thymus undergoes an age‐related progressive atrophy called involution that is characterized by qualitative and quantitative changes in stromal cells as well as their replacement with adipocytes.^3^, ^139^, ^140^ In particular, mTECs exhibit a marked decrease in cellularity and an alteration in gene expression patterns with aging.^46^, ^141^ In contrast, the frequency of thymic fibroblasts increases with aging, so the ratio of fibroblasts to TECs is markedly increased in aged mice.^46^ It was shown that TECs in aged mice can give rise to fibroblasts and further into adipocytes, by a process called epithelial‐to‐mesenchymal transition (EMT).^142^, ^143^ This suggests the possibility that a fraction of thymic fibroblasts may be of TEC origin. However, fate‐mapping studies using TEC‐specific Cre lines (Psmb11‐Cre or FoxN1‐Cre) demonstrated that only a small percentage of fibroblasts (up to approximately 10%) may be derived from the TEC lineage, and these cells do not increase with age.^5^, ^72^ Therefore, the majority of thymic fibroblasts in the adult thymus is indeed NC‐derived, so the contribution of EMT to the development of thymic fibroblasts, if there is any, is limited.

Ucar et al reported that the thymic stroma in adult mice contains cells that under low‐attachment culture conditions form spheres (termed thymospheres),^144^ and Sheridan et al subsequently demonstrated that those cells do not belong to the TEC lineage but rather, contain mesenchymal stem cells.^145^ These thymosphere‐forming cells were shown to be capable of giving rise to fibroblasts and adipocytes under appropriate culture conditions. It was also shown in an early study that mesenchymal stromal cells isolated from human thymus are able to differentiate in vitro into adipocytes.^146^ Nevertheless, at present, there is no conclusive evidence as to which TECs or thymic fibroblasts are responsible for the age‐related adiposis of the thymus. This is an important issue for understanding the contribution of the entire repertoire of thymic stromal cells, including fibroblasts, to age‐related thymic atrophy, as well as for exploring the possible technologies that would allow thymic regeneration.

## 6 CONCLUDING REMARKS

With the recent advance of large‐scale datasets of stromal cells across multiple organs, we now stand at a new beginning for a comprehensive understanding of cellular characteristics and interactions in the immune system. Such bioinformatics approaches, along with certain long‐sought results in histology and embryology, have unveiled the versatile range of functions of thymic fibroblasts in supporting thymus organogenesis and T cell development. Efforts to understand thymic fibroblast function are now being applied to the studies of human thymus, which may shed light on the role of thymic fibroblasts in human health and disease. In particular, medullary fibroblasts are an emerging subset of thymic stromal cells that is essential for the self‐antigen expression to induce immune tolerance. This finding also suggests an intrinsic need for the thymic medulla to embrace a variety of cell types, each of which expresses cell type‐restricted antigens in order to produce the diverse array of self‐antigens required to accomplish T cell selection. A major issue that remains to be addressed in future is the cellular and molecular basis for fibroblast cooperation with other stromal cells in age‐related thymic atrophy as well as autoimmunity. Elucidating the lineage relationships and cell‐cell interactions of stromal cells as well as their significance in TCR repertoire formation in the degenerating thymus will open up possibilities to better understand and control the thymic microenvironment in future therapeutic applications.

## CONFLICT OF INTEREST

There is no conflict of interest to declare.
